# The role of FLI-1-EWS, a fusion gene reciprocal to EWS-FLI-1, in Ewing sarcoma

**DOI:** 10.18632/genesandcancer.86

**Published:** 2015-11

**Authors:** David J. Elzi, Meihua Song, Peter J. Houghton, Yidong Chen, Yuzuru Shiio

**Affiliations:** ^1^ Greehey Children's Cancer Research Institute, The University of Texas Health Science Center, San Antonio, Texas, USA; ^2^ Cancer Therapy and Research Center, The University of Texas Health Science Center, San Antonio, Texas, USA; ^3^ Department of Molecular Medicine, The University of Texas Health Science Center, San Antonio, Texas, USA; ^4^ Department of Epidemiology and Biostatistics, The University of Texas Health Science Center, San Antonio, Texas, USA; ^5^ Department of Biochemistry, The University of Texas Health Science Center, San Antonio, Texas, USA

**Keywords:** EWS-FLI-1, Ewing sarcoma, FLI-1-EWS

## Abstract

Ewing sarcoma is a cancer of bone and soft tissue in children that is characterized by a chromosomal translocation involving EWS and an Ets family transcription factor, most commonly FLI-1. The EWS-FLI-1 fusion oncogene is widely believed to play a central role in Ewing sarcoma. The EWS-FLI-1 gene product regulates the expression of a number of genes important for cancer progression, can transform mouse cells such as NIH3T3 and C3H10T1/2, and is necessary for proliferation and tumorigenicity of Ewing sarcoma cells, suggesting that EWS-FLI-1 is the causative oncogene. However, a variety of evidence also suggest that EWS-FLI-1 alone cannot fully explain the Ewing sarcomagenesis.

Here we report that FLI-1-EWS, a fusion gene reciprocal to EWS-FLI-1, is frequently expressed in Ewing sarcoma. We present evidence suggesting that endogenous FLI-1-EWS is required for Ewing sarcoma growth and that FLI-1-EWS cooperates with EWS-FLI-1 in human mesenchymal stem cells, putative cells of origin of Ewing sarcoma, through abrogation of the proliferation arrest induced by EWS- FLI-1.

## INTRODUCTION

Ewing sarcoma is an aggressive cancer of bone and soft tissues in children with poor long-term outcome. Ewing sarcoma is characterized by the reciprocal chromosomal translocation generating a fusion oncogene between EWS and an Ets family transcription factor, most commonly FLI-1 [1-5]. EWS-FLI-1 translocation accounts for 85% of Ewing sarcoma cases.

Since the cloning of the EWS-FLI-1 fusion oncogene [6], the predominant view in the Ewing sarcoma field has been that EWS-FLI-1 plays a central role in Ewing sarcomagenesis [1-5]. The EWS-FLI-1 gene product regulates the expression of a number of genes important for cancer progression [7], can transform mouse cells such as NIH3T3 [8] and C3H10T1/2 [9], and is necessary for proliferation and tumorigenicity of Ewing sarcoma cells [1-5], suggesting that EWS-FLI-1 is the causative oncogene.

However, a variety of evidence also suggest that EWS-FLI-1 alone cannot fully explain the Ewing sarcomagenesis: 1) EWS-FLI-1 alone cannot transform any human cell types including mesenchymal stem cells (MSCs) which are the putative cells of origin of Ewing sarcoma [1-4]; 2) Generating a transgenic mouse model of Ewing sarcoma by using EWS-FLI-1 alone has been unsuccessful [1-3]; and 3) Other genetic alterations such as mutations of INK4a and p53, although far less common than EWS-FLI-1 translocation, confer worse clinical outcome [1, 2].

**Figure 1:**
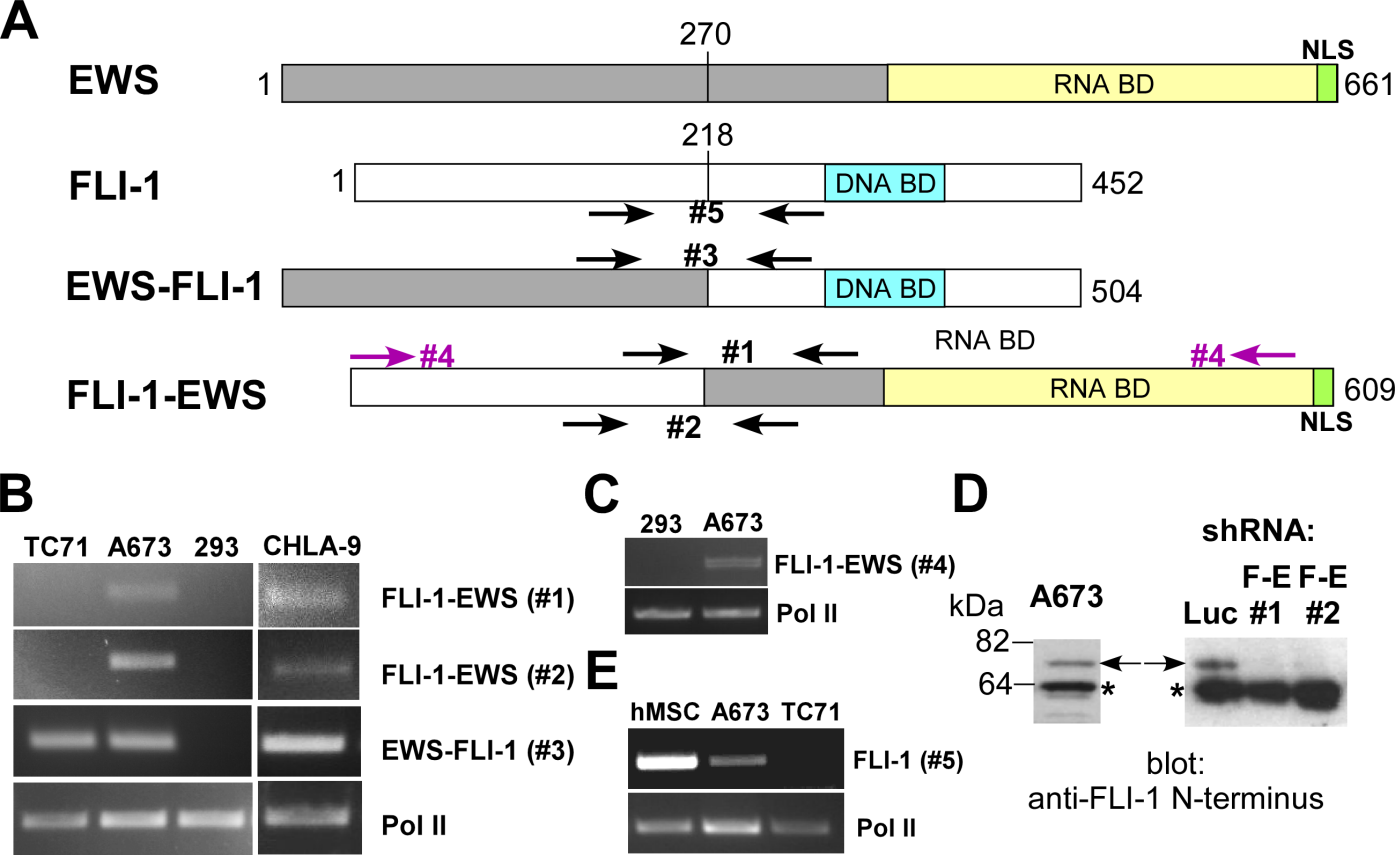
Expression of FLI-1-EWS in Ewing sarcoma cells (A) Structure of EWS, FLI-1, EWS-FLI-1, and FLI-1-EWS, and PCR primers used. (B) Expression of FLI-1-EWS in A673 and CHLA-9 Ewing sarcoma cells. FLI-1-EWS or EWS-FLI-1 was amplified using the indicated PCR primers. RNA polymerase II (Pol II) serves as a loading control. (C) Detection of nearly full-length FLI-1-EWS in A673 cells. (D) Endogenous FLI-1-EWS protein expression in A673 cells. A673 cells were infected with lentiviruses expressing shRNAs against FLI-1-EWS or luciferase and were selected with 2 μg/ml puromycin for 2 days. The FLI-1-EWS protein expression was examined by anti-FLI-1 N-terminus antibody immunoblotting. Arrows denote the Fli-1-EWS protein which was silenced by specific shRNAs (FLI- 1-EWS shRNA-1 and 2). Luciferase shRNA serves as a control. Asterisks denote non-specific protein bands. (E) Expression of the FLI-1 mRNA in A673 cells and human mesenchymal stem cells.

Recent genome sequencing studies confirmed the long-held view that EWS-FLI-1 translocation is the only recurrent genetic alteration commonly found in Ewing sarcoma [10-12]. This chromosomal translocation generates two fusion genes, EWS-FLI-1 and FLI-1-EWS (Figure 1A). Previous research has been centered on EWS- FLI-1, which is considered as the main oncogenic driver of Ewing sarcoma. In contrast, the reciprocal fusion gene, FLI-1-EWS, has not been studied because the attempts to detect FLI-1-EWS mRNA expression in Ewing sarcoma mainly by Northern blotting were not successful [6, 13, 14] and the translocated chromosome encoding FLI-1- EWS can be lost secondarily in a small subset of Ewing sarcoma cells [15].

We have now discovered that FLI-1-EWS is frequently expressed in Ewing sarcoma and have obtained data indicating that endogenous FLI-1-EWS is required for Ewing sarcoma growth and that FLI-1-EWS cooperates with EWS-FLI-1 in human MSCs through abrogation of the growth arrest induced by EWS-FLI-1.

## RESULTS & DISCUSSION

### FLI-1-EWS expression in Ewing sarcoma

The reciprocal chromosomal translocation between EWS and FLI-1 in Ewing sarcoma generates two fusion genes, EWS-FLI-1 and FLI-1-EWS (Figure 1A). Although previous studies failed to detect the expression of FLI-1- EWS in Ewing sarcoma by Northern blotting [6, 13, 14], we became interested in the possibility that FLI-1-EWS *is* expressed in some Ewing sarcoma cells or *was* expressed when the EWS-FLI-1 chromosomal translocation occurred in the Ewing sarcoma cell of origin. Using two different pairs of PCR primers (#1 and #2, see Figure 1A), we were able to detect the FLI-1-EWS fusion transcript in A673 and CHLA-9, but not in TC71 Ewing sarcoma cells (Figure 1B and Supplementary Table S1; Note the primers used for PCR amplification span a 9-kb intron in FLI-1 and the genomic locus cannot be amplified; RNA polymerase II (Pol II) serves as a loading control; Human kidney 293 cells serve as negative control). The EWS-FLI-1 fusion transcript (type 1) was detectable in A673, CHLA-9, and TC71, but not in 293 (Figure 1B and Supplementary Table S1).

Furthermore, by optimizing the RT-PCR conditions for longer transcripts, we were able to amplify nearly full-length FLI-1-EWS ORF (including the initiation codon, using primer pair #4, Figure 1C) from A673 cellular RNA. We went on to clone the nearly full-length FLI- 1-EWS ORF amplified from A673 cells and verified its entire DNA sequence as FLI-1-EWS, unequivocally proving the existence of FLI-1-EWS transcript in A673 cells. Importantly, we were able to detect endogenous FLI-1-EWS protein (~75 kDa) in A673 cells by anti- FLI-1 N-terminus antibody immunoblotting, which was silenced by two shRNAs that target the junction of FLI-1 and EWS in FLI-1-EWS (Figure 1D; Note these shRNAs also silenced FLI-1-EWS mRNA, see Figure 3A). We also analyzed the expression of FLI-1 using primers specific to un-translocated FLI-1 (#5 in Figure 1A) and detected FLI-1 mRNA expression in A673, but not in TC71 cells (Figure 1E). Interestingly, human primary mesenchymal stem cells (MSCs), the putative cells of origin of Ewing sarcoma [1-4], expressed higher levels of FLI-1 than A673 cells (Figure 1E and Supplementary Table S2). This indicates that the FLI-1 gene promoter is active in MSCs and suggests that FLI-1-EWS would be expressed if EWS- FLI-1 translocation occurs in MSCs.

**Figure 2:**
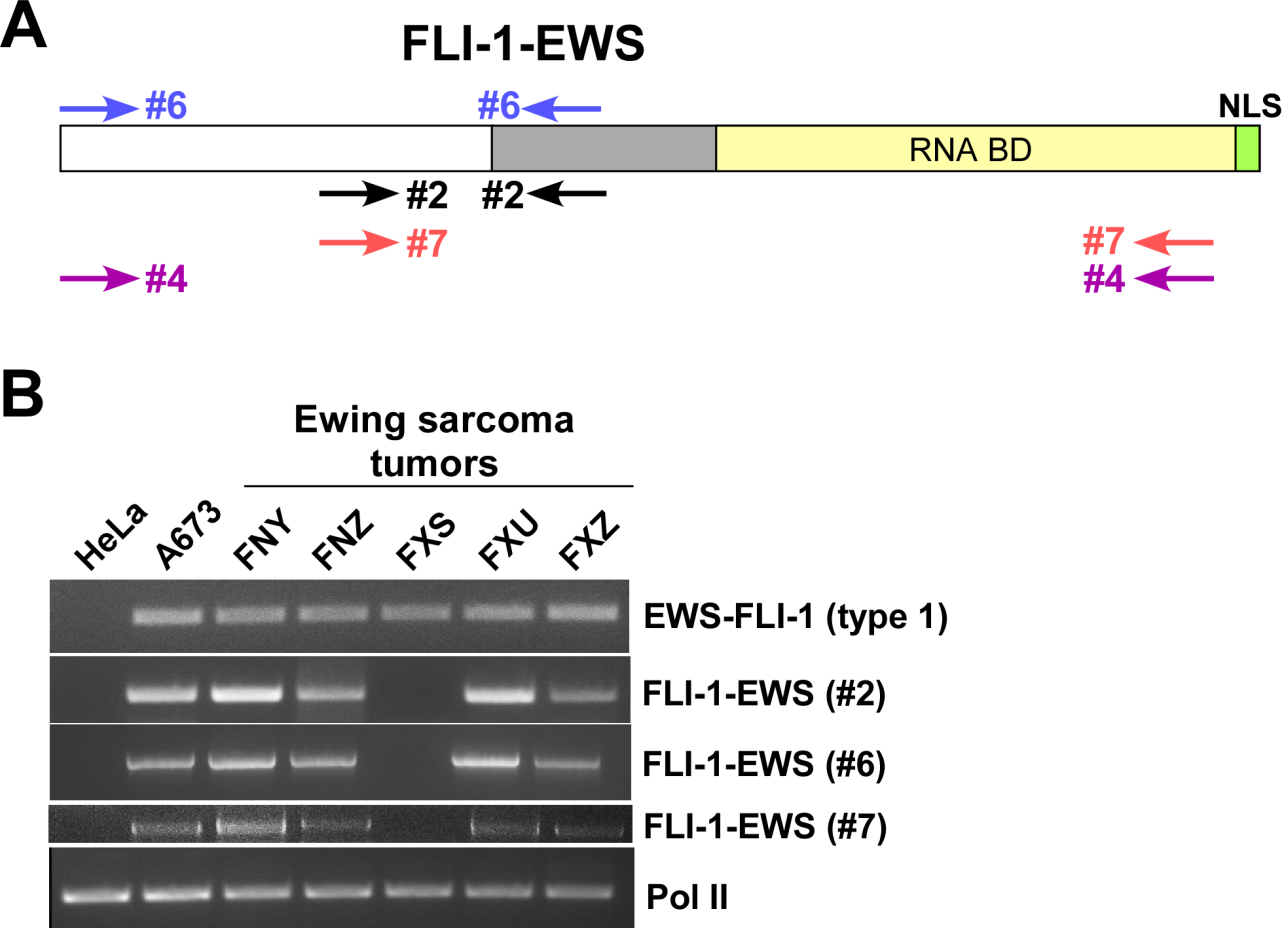
Expression of FLI-1-EWS in Ewing sarcoma tumors (A) PCR primers used for FLI-1-EWS amplification. (B) FLI-1-EWS and EWS-FLI-1 expression in Ewing sarcoma tumors.

As an initial screen for the prevalence of FLI-1- EWS expression in Ewing sarcoma tumors, we obtained Ewing sarcoma tumor RNA samples from the Cooperative Human Tissue Network and analyzed the expression of FLI-1-EWS. Of the five tumors expressing EWS-FLI-1 fusion transcript (type 1 fusion), four tumors expressed FLI-1-EWS (Figure 2 and Supplementary Table S3). Using the primer pair #4 (Figure 1A and 2A), we amplified nearly full-length FLI-1-EWS ORF from case FNY RNA sample and verified its entire DNA sequence as FLI-1- EWS, proving the existence of FLI-1-EWS transcript in Ewing sarcoma tumor. These results suggest that FLI-1- EWS expression is prevalent in Ewing sarcoma cell lines and tumors.

### FLI-1-EWS makes a positive contribution to Ewing sarcoma growth

**Figure 3:**
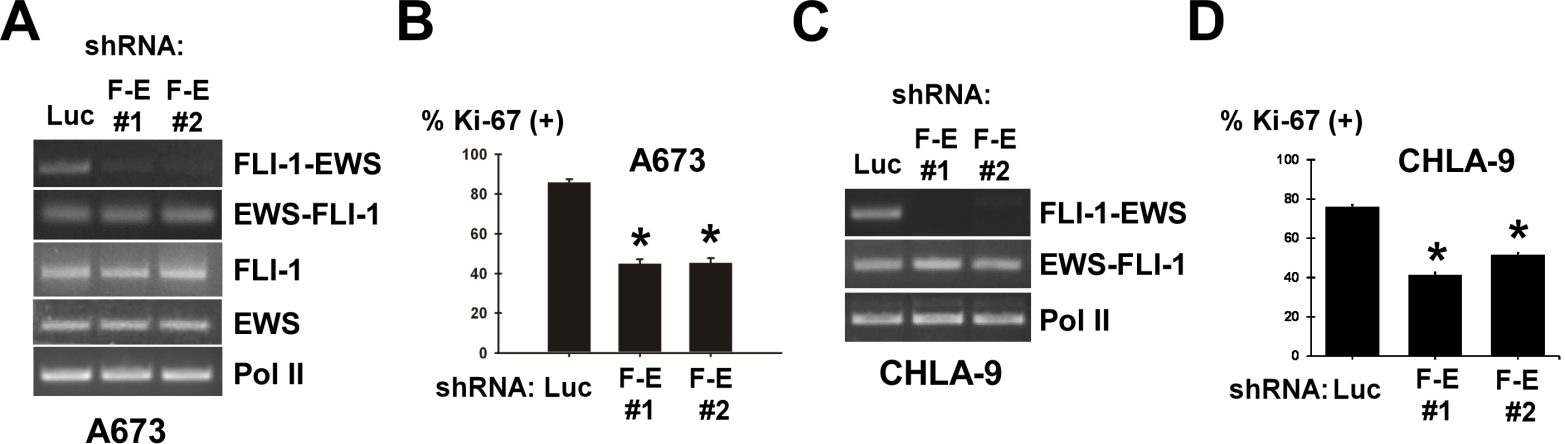
FLI-1-EWS silencing inhibits Ewing sarcoma proliferation (A) Silencing of FLI-1-EWS (F-E) in A673 cells by shRNAs. A673 cells were infected with lentiviruses expressing shRNAs against FLI-1-EWS or luciferase and were selected with 2 μg/ ml puromycin for 2 days. The expression of FLI-1-EWS, EWS-FLI-1, FLI-1, EWS, and RNA polymerase II was examined by RT-PCR. (B) FLI-1-EWS knockdown inhibits A673 cell proliferation. Cell proliferation was assessed by Ki-67 staining. Asterisks denote p < 0.05 compared with control luciferase shRNA. (C) Silencing of FLI-1-EWS in CHLA-9 cells by shRNAs. CHLA-9 cells were infected with lentiviruses expressing shRNAs against FLI-1-EWS or luciferase and were selected with 2 μg/ml puromycin for 2 days. The expression of FLI-1-EWS, EWS-FLI-1, and RNA polymerase II was examined by RT-PCR. (D) FLI-1-EWS knockdown inhibits CHLA-9 cell proliferation. Cell proliferation was assessed by Ki-67 staining. Asterisks denote p < 0.05 compared with control luciferase shRNA.

To gain an insight into the function of FLI-1-EWS in Ewing sarcoma cells, we employed two shRNAs that target the junction of FLI-1 and EWS in FLI-1-EWS and expressed these shRNAs in A673 and CHLA-9 Ewing sarcoma cells using lentiviral vectors, which resulted in the silencing of FLI-1-EWS (Figure 3A, 3C, and 1D; luciferase shRNA-expressing virus serves as control; Note FLI-1-EWS shRNAs did not affect the expression of EWS-FLI-1, FLI-1, or EWS) and significant inhibition of proliferation as determined by Ki-67 staining (Figure 3B and 3D; asterisks denote p< 0.05 compared to luciferase shRNA control). This demonstrates that endogenous FLI- 1-EWS makes a positive contribution to Ewing sarcoma cell proliferation.

**Figure 4:**
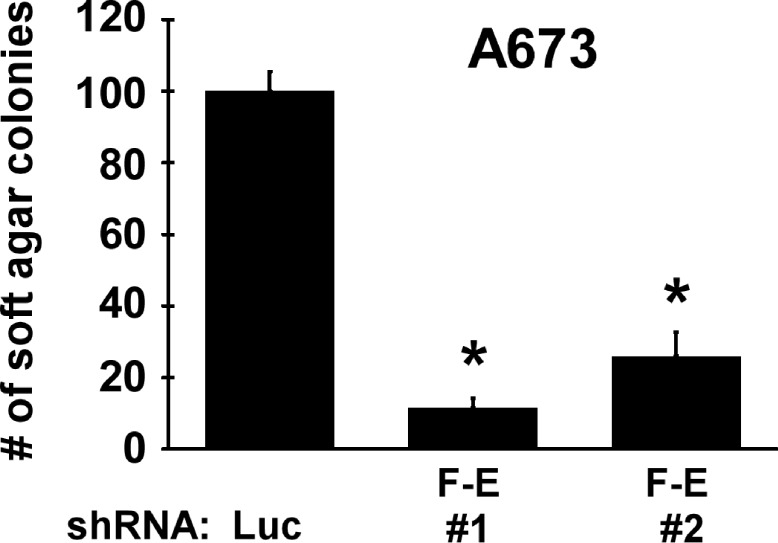
FLI-1-EWS is essential for anchorage-independent growth of Ewing sarcoma cells A673 cells were infected with lentiviruses expressing shRNAs against FLI-1-EWS or luciferase and were selected with 2 μg/ml puromycin for 2 days. Four days after infection, cells were plated in semi-solid medium. One week later, colonies were counted. Asterisks denote p < 0.05 compared with control luciferase shRNA.

One of the hallmarks of cancer is the ability to proliferate independent of anchorage. Importantly, silencing of FLI-1-EWS in A673 cells resulted in dramatic inhibition of soft agar colony formation (Figure 4), indicating that FLI-1-EWS plays an essential role in anchorage-independent growth of Ewing sarcoma cells.

**Figure 5:**
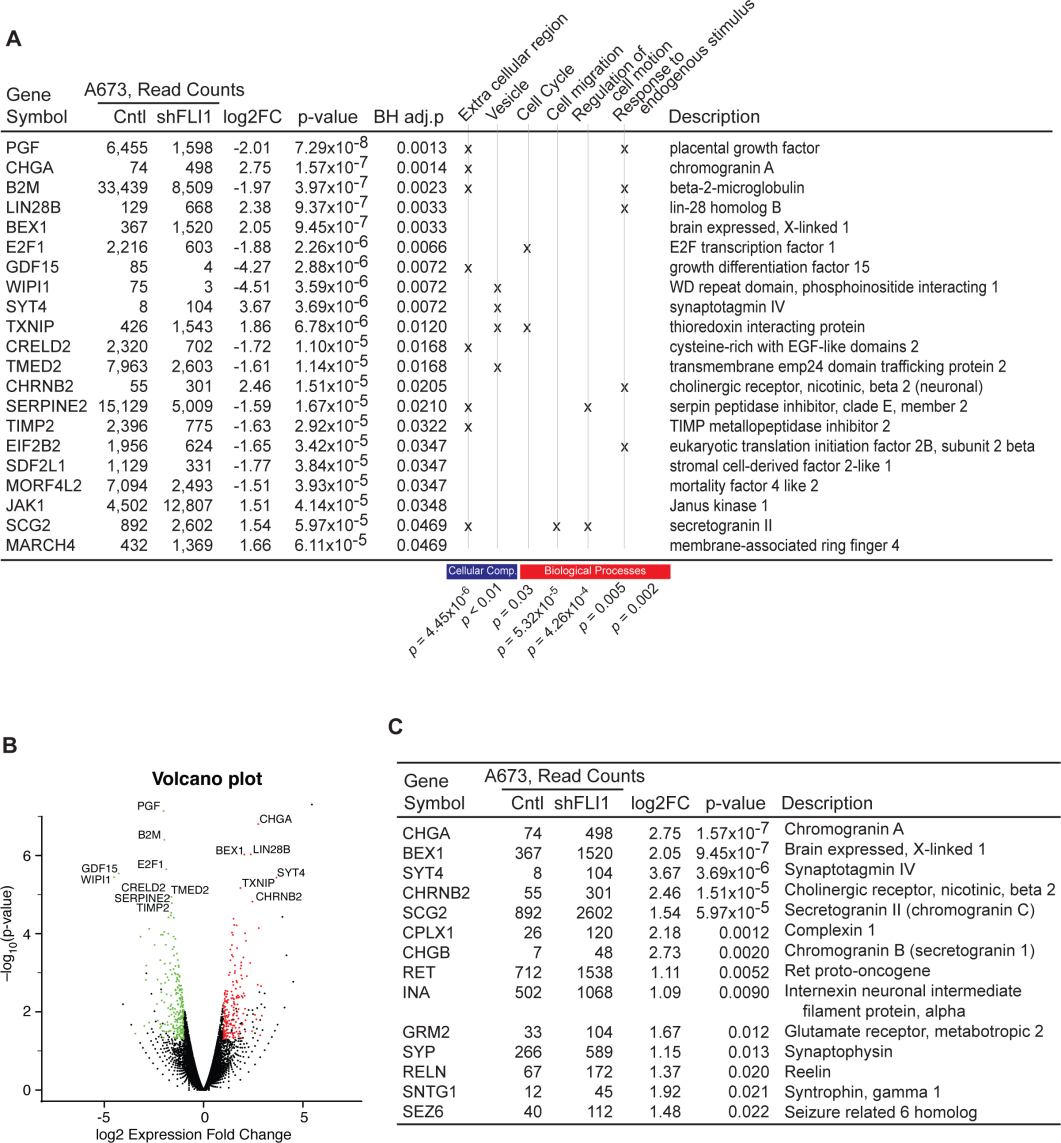
Gene expression changes in FLI-1-EWS-silenced A673 cells A673 cells were infected with lentiviruses expressing shRNA against FLI-1-EWS or luciferase and were selected with 2 μg/ml puromycin for 2 days. Four days after infection, total RNA was isolated and global gene expression was analyzed by RNA-sequencing. Differential gene expression analysis was carried out using DESeq software with sequence read counts for each gene evaluated using HTSeq (see Materials & Methods). (A) Twenty one genes whose adjusted *p*-value < 0.05 (Benjamini-Hochberg correction for multiple test), absolute log2 fold-change > 1, average expression level of control and FLI-1-EWS knockdown > 10, and RPKM > 1 are listed. 419 genes with fold-change > 2 (or absolute log2 fold-change > 1) were selected and submitted to DAVID (see Materials & Methods) for Gene Ontology enrichment analysis. Top six functional clusters with score > 2 were selected. Genes with a specific function are marked with “x”. The enrichment *p*-value is provided at the bottom. Read counts listed in the table are normalized read counts provided by DESeq. (B) Volcano plot of all genes, with upregulated genes marked in red and downregulated genes in green. (C) List of neural genes induced in FLI-1-EWS-sileneced A673 cells.

To further dissect the biological role of FLI-1- EWS, global mRNA expression changes induced by FLI- 1-EWS silencing were analyzed by RNA-sequencing (Figure 5 and Supplementary Table S4). Interestingly we found upregulation of a number of neural genes in FLI- 1-EWS-silenced A673 cells (Figure 5C). Ewing sarcoma has a tendency for neural differentiation in response to a variety of stimuli including cAMP, TPA, retinoic acid, and Wnt signaling [16-18]. Induction of neural genes upon FLI-1-EWS silencing might suggest that FLI-1-EWS prevents neural differentiation of Ewing sarcoma cells and maintains their continuous proliferation.

**Figure 6:**
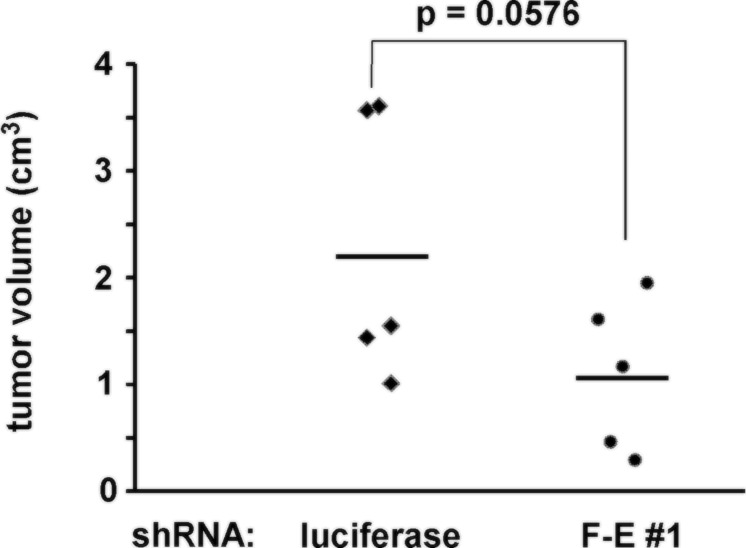
The effect of FLI-1-EWS silencing on Ewing sarcoma tumorigenicity A673 cells were infected with lentiviruses expressing FLI-1-EWS shRNA-1 (F-E #1) or luciferase shRNA and were selected with 2 μg/ml puromycin for 2 days. Two million cells were subcutaneously injected into SCID mice and four weeks after injection, tumor volume was determined using a caliper (5 mice/group).

To test the role of FLI-1-EWS in the tumorigenicity of Ewing sarcoma, we employed xenograft tumorigenicity assays in SCID mice. A673 cells were infected with lentiviruses expressing shRNA against FLI-1-EWS or luciferase. After puromycin selection, cells were subcutaneously injected into the flanks of SCID mice. Tumor volume was determined four weeks after injection. As shown in Figure 6, FLI-1-EWS silencing reduced xenograft tumor growth (p = 0.0576).

These results indicate that endogenous FLI-1-EWS makes a positive contribution to Ewing sarcoma growth.

### FLI-1-EWS and EWS-FLI-1 cooperate in mesenchymal stem cells

**Figure 7:**
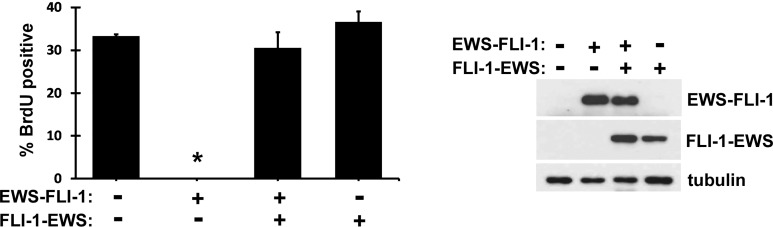
FLI-1-EWS abrogates the proliferation arrest induced by EWS-FLI-1 in mesenchymal stem cells Human mesenchymal stem cells were infected with lentiviruses expressing EWS-FLI-1 and/or FLI-1-EWS. Two days after infection, cells were selected with 2 μg/ml puromycin and 150 μg/ml hygromycin for 2 days. Cell proliferation was assessed by BrdU incorporation (left). Asterisk denotes p < 0.05 compared with EWS-FLI-1/FLI-1-EWS co-expressing cells as well as vector-expressing cells. The expression of EWS-FLI-1 and FLI-1-EWS was examined by immunoblotting with tubulin as a loading control (right).

Although EWS-FLI-1 is considered an oncogene, its expression often displays toxicity: Expression of EWS-FLI-1 is known to potently inhibit the proliferation of primary human and mouse fibroblasts; transgenic expression of EWS-FLI-1 in mice results in lethality, which is considered one of the major obstacles to the development of a mouse model of Ewing sarcoma. To test whether FLI-1-EWS cooperates with EWS-FLI-1 by alleviating the toxicity of EWS-FLI-1, we co-expressed FLI-1-EWS and EWS-FLI-1 in human mesenchymal stem cells (MSCs), putative cells of origin of Ewing sarcoma. Human MSCs (derived from cord blood, purchased from Vitro BioPharma) were infected with lentiviruses expressing FLI-1-EWS, EWS-FLI-1, or both (empty vector as control). We reproducibly observed the proliferation arrest of human MSCs by EWS-FLI-1, which was abolished by the co-expression of FLI-1-EWS (Figure 7, proliferation assessed by BrdU incorporation). The expression of FLI-1-EWS and EWS-FLI1 was verified by immunoblotting (Figure 7) and quantitative RT-PCR (Supplementary Table S5). The abrogation of EWS-FLI- 1-induced proliferation arrest by FLI-1-EWS in human MSCs raises an important possibility that these two fusion genes generated by reciprocal chromosomal translocation cooperate in the cells of origin of Ewing sarcoma and together drive tumor initiation.

Chromosomal translocation is frequently associated with sarcomas and hematological malignancies and often generates two fusion genes. Typically, one of the two fusion genes is invariably expressed in tumors, displays oncogenic property, and is hence intensively studied as a main oncogenic driver whereas the reciprocal fusion gene receives much less attention for reasons including variable expression in established tumors, lack of oncogenic property, and lack of major functional domains encoded by the parental genes. Accumulating evidence, however, suggests that reciprocal fusion genes can contribute to tumorigenesis and disease phenotype in conjunction with their reciprocal oncogenic fusion partners [19-24]. A common theme emerged from these studies is that the biological roles of reciprocal fusion genes need to be more carefully examined, given the limitation of available experimental tools to study tumor initiation process in humans.

Here we provide several lines of evidence that FLI-1-EWS, a fusion gene reciprocal to EWS-FLI-1, is frequently expressed in Ewing sarcoma and contributes to tumor cell growth. FLI-1-EWS abrogated the toxicity displayed by EWS-FLI-1 in human MSCs, which is in principle consistent with the notion that the two fusion genes cooperate at the early stage of Ewing sarcomagenesis. Although the EWS-FLI-1 transcript, which is driven by the gene promoter for highly expressed EWS [25], was one to two orders of magnitude more abundant than the FLI-1-EWS transcript in Ewing sarcoma cell lines and tumors (Supplementary Table S1 and S3), silencing FLI-1-EWS nonetheless impaired anchorage-dependent and anchorage-independent growth as well as tumorigenicity of Ewing sarcoma, indicating that FLI-1-EWS plays an important role in this tumor. Furthermore, we found that the FLI-1 transcript levels are several hundred-fold higher in human MSCs than in A673 Ewing sarcoma cells (Supplementary Table S2), suggesting that a considerable amount of FLI-1- EWS transcript could be expressed when EWS-FLI-1 translocation occurred in MSCs, putative Ewing sarcoma cells of origin. It is possible that high levels of FLI-1-EWS contribute to the initiation of Ewing sarcoma while the FLI-1-EWS expression declines during tumor progression, which might explain the lack of FLI-1-EWS expression in some Ewing sarcoma cells such as TC71 (Figure 1B).

Whereas FLI-1-EWS knockdown inhibited the anchorage-dependent and anchorage-independent growth of Ewing sarcoma and FLI-1-EWS cooperated with EWS- FLI-1 in mesenchymal stem cells, we found that FLI-1- EWS itself is not sufficient to transform mouse NIH3T3 cells and that FLI-1-EWS alone is not sufficient to make cells proliferate independent of anchorage. These findings suggest the possibility that FLI-1-EWS acts in cooperation with EWS-FLI-1 to drive Ewing sarcomagenesis.

The molecular mechanism of FLI-1-EWS action is not yet clear. One possibility is that FLI-1-EWS sequesters certain RNAs through its RNA-binding domain (Figure 1A). EWS and its paralogues, FUS and TAF15, harbor prion-like low-complexity domains at their N-termini which could form higher-order fibrous assemblies [26-29]. Because FLI-1-EWS lacks the N-terminal low-complexity domain, the RNAs bound by FLI-1-EWS may not be recruited to the postulated fibrous assemblies containing EWS. Alternatively, FLI-1-EWS might have a gain of function as is the case for EWS-FLI-1. The C-terminal portion of EWS, FUS, and TAF15 was recently shown to function as a sensor of poly(ADP-ribose) [30]. EWS, FUS, and TAF15 mediated poly(ADP-ribose)-seeded liquid de- mixing and assembly of intrinsically disordered proteins at the sites of DNA damage [30]. FLI-1-EWS might similarly sense poly(ADP-ribose) using its C-terminal domain and recruit the N-terminal transcriptional activation domain of FLI-1 to the sites of poly(ADP-ribose) accumulation, altering gene expression. To address how FLI-1-EWS functions, it will be important to characterize the protein interactors for FLI-1-EWS. It will also be important to test whether the cooperation between FLI-1-EWS and EWS- FLI-1 can be used to develop an animal model of Ewing sarcoma. Future work should clarify the precise role of FLI-1-EWS in Ewing sarcomagenesis.

## MATERIALS AND METHODS

### Cell culture

293 and 293T cells were cultured in Dulbecco's modified Eagle's medium (DMEM) supplemented with 10% calf serum. A673 cells were cultured in DMEM supplemented with 10% fetal calf serum. CHLA-9 cells and TC71 cells were cultured in RPMI1640 medium supplemented with 10% fetal calf serum. Cord blood- derived human mesenchymal stem cells were purchased from Vitro Biopharma (Golden, CO) and cultured in low serum MSC-GRO following the manufacturer's procedure. Calcium phosphate co-precipitation was used for transfection of 293T cells. Lentiviruses were prepared by transfection in 293T cells following System Biosciences' protocol and the cells infected with lentiviruses were selected with 2 μg/ml puromycin for 48 hours as described [31, 32]. For co-infection of human mesenchymal stem cells, the infected cells were selected with 2 μg/ml puromycin and 150 μg/ml hygromycin for 48 hours. FLI-1-EWS cDNA (see Figure 1A) was cloned into pCDH1-puro lentiviral vector (System Biosciences) and EWS-FLI-1 cDNA was cloned into a modified pCDH1 vector with hygromycin resistance marker. The target sequences for shRNAs are as follows: FLI-1-EWS shRNA-1, GAGTGTCAAAGAAGGTTCATT; FLI-1- EWS shRNA-2, CAAAGAAGGTTCATTCCGACA; and luciferase shRNA, GCACTCTGATTGACAAATACGATTT. The shRNAs were expressed using pSIH-H1-puro lentiviral vector (System Biosciences).

### RT-PCR

Total cellular RNA was isolated using TRIzol reagent (Invitrogen) and RT-PCR was performed as described previously [31, 32], using GoTaq DNA polymerase or GoTaq Hot Start polymerase (Promega). The PCR cycle numbers were FLI-1-EWS (40), EWS-FLI-1 (25), FLI-1 (40), EWS (25), and RNA polymerase II (25). The following primers were used (see Figure 1A for the location of primers): FLI-1- EWS #1 5′ primer, AATACAACCTCCCACACCGA, 3′ primer, ACTCCTGCCCATAAACACCC; FLI-1- EWS #2 5′ primer, GTGCTGTTGTCACACCTCAG, 3′ primer, GTTCTCTCCTGGTCCGGAAA; EWS- FLI-1 #3 5′ primer, GCACCTCCATCCTACCCTCCT, 3′ primer, TGGCAGTGGGTGGGTCTTCAT; Fli-1- EWS #4, 5′ primer, ATGGACGGGACTATTAAGGA, 3′ primer, CTCGTCTTCCTCCACCAAAG; Fli-1 #5, 5′ primer, AATACAACCTCCACACCGA, 3′ primer, CTTACTGATCGTTTGTGCCCC; Fli-1- EWS #6, 5′ primer, ATGGACGGGACTATTAAGGA, 3′ primer, GTTCTCTCCTGGTCCGGAAA; Fli-1- EWS #7 5′ primer, GTGCTGTTGTCACACCTCAG, 3′ primer, CTCGTCTTCCTCCACCAAAG; EWS 5′ primer, CAGCCTCCCACTAGTTACCC, 3′ primer, GTTCTCTCCTGGTCCGGAAA; and RNA polymerase II (Pol II) 5′ primer, GGATGACCTGACTCACAAACTG, 3′ primer, CGCCCAGACTTCTGCATGG. The quantitative real-time RT-PCR was performed using GoTaq® qPCR Master Mix (Promega) and 7500 Real-Time PCR System (Applied Biosystems). FLI1-EWS #2 primers were used.

### Immunoblotting and immunofluorescence

Immunoblotting and immunofluorescence were performed as described [31, 32]. The following antibodies were used: mouse monoclonal anti-BrdU (BD Pharmingen); sheep polyclonal anti-FLI-1 (AF6474, R&D Systems); rabbit polyclonal anti-FLI-1 (ab15289, Abcam); mouse monoclonal anti-Ki-67 (BD Pharmingen); and mouse monoclonal anti-tubulin (DM1A, Sigma-Aldrich).

### RNA sequencing

A673 cells were infected with lentiviruses expressing FLI-1-EWS shRNA-1 or luciferase shRNA and were selected with 2 μg/ml puromycin. Four days after infection total RNA was isolated using TRIzol reagent (Invitrogen). RNA quality was assessed by Bioanalyzer and poly A(+) RNA was isolated by oligo-dT purification and fragmented using divalent cations under elevated temperature. cDNA fragment libraries were synthesized following the TruSeq mRNA-seq Library Preparation protocol (Illumina, San Diego, CA). We obtained 24.3 and 20.2 million sequence reads for control and FLI-1-EWS knockdown samples, respectively, using Illumina HiSeq system at the Greehey Children's Cancer Research Institute Genome Sequencing Facility, employing a 50bp single-read sequencing protocol.

Sequence reads were first aligned with TopHat [33] to human genome (NCBI GRCh37/UCSC hg19), allowing no more than 2 mismatches in the alignment. After alignment, reads aligned to known transcripts were counted using HTSeq [34]. Expression abundance of each gene was evaluated by a unit of read count and RPKM (read per kilobase of transcript per million reads mapped). Differential gene expression was calculated using DESeq [35] to obtain fold-change, *p*-value, and *p*-value adjusted by Benjamini-Hochberg correction for multiple tests [36]. We selected differentially expressed genes based on the following criteria: 1) fold-change > 2 (and adjusted *p*-value < 0.05) and 2) RPKM > 1. Functional assessment of these differentially expressed genes was performed by using Database for Annotation, Visualization and Integrated Discovery (DAVID, http://david.abcc.ncifcrf.gov/) [37] and Ingenuity pathway analysis (IPA, Ingenuity Systems, http://www.ingenuity.com).

### Soft agar colony formation assays

A673 cells were infected with lentiviruses expressing shRNAs against FLI-1-EWS or luciferase and were selected with 2 μg/ml puromycin. Four days after infection, 1×10^3^ cells were plated in soft agar. The soft agar cultures were comprised of two layers: a base layer (2 ml/well in a 6-well plate; DMEM/10% fetal calf serum and 1.2% agarose) and a cell layer (2 ml/well in a 6-well plate; DMEM/10% fetal calf serum and 0.6% agarose). Colonies were grown for one week and counted.

### Xenograft tumorigenicity assays

A673 cells were infected with lentiviruses expressing FLI-1-EWS shRNA-1 or luciferase shRNA and were selected with 2 μg/ml puromycin for 2 days. Each cell type was subcutaneously injected into the flanks of SCID mice (2×10^6^ cells/injection, n=5). Tumor growth was monitored weekly using a caliper.

### Tumor RNA samples

De-identified Ewing sarcoma tumor RNA samples were obtained from the Cooperative Human Tissue Network.

## SUPPLEMENTARY TABLES


